# Rethinking Models of Outpatient Specialist Care in Type 2 Diabetes Using eHealth: Study Protocol for a Pilot Randomised Controlled Trial

**DOI:** 10.3390/ijerph16060959

**Published:** 2019-03-18

**Authors:** Anish Menon, Farhad Fatehi, Dominique Bird, Darsy Darssan, Mohan Karunanithi, Anthony Russell, Leonard Gray

**Affiliations:** 1 Centre for Health Services Research, Faculty of Medicine, Brisbane, The University of Queensland, QLD 4102, Australia; f.fatehi@uq.edu.au (F.F.); d.bird@uq.edu.au (D.B.); len.gray@uq.edu.au (L.G.); 2 Department of Diabetes and Endocrinology, Princess Alexandra Hospital, Woolloongabba, Brisbane, QLD 4102, Australia; Anthony.Russell2@health.qld.gov.au; 3 CSIRO Australian e-Health Research Centre, Brisbane, QLD 4102, Australia; Mohan.Karunanithi@csiro.au; 4 School of Advanced Technologies in Medicine, Tehran University of Medical Sciences, Tehran 14174, Iran; 5 School of Public Health, The University of Queensland, Brisbane, QLD 4006, Australia; d.darssan@uq.edu.au; 6 Faculty of Medicine, The University of Queensland, Brisbane, QLD 4102, Australia

**Keywords:** diabetes, models of care, insulin dose adjustment, mHealth, eHealth, digital health, telemedicine, telehealth

## Abstract

Conventional outpatient services are unlikely to meet burgeoning demand for diabetes services given increasing prevalence of diabetes, and resultant impact on the healthcare workforce and healthcare costs. Disruptive technologies (such as smartphone and wireless sensors) create an opportunity to redesign outpatient services. In collaboration, the Department of Diabetes and Endocrinology at Brisbane Princess Alexandra Hospital, the University of Queensland Centre for Health Services Research and the Australian e-Health Research Centre developed a mobile diabetes management system (MDMS) to support the management of complex outpatient type 2 diabetes mellitus (T2DM) adults. The system comprises of a mobile App, an automated text-messaging feedback and a clinician portal. Blood glucose levels (BGL) data are automatically transferred by Bluetooth-enabled glucose meter to the clinician portal via the mobile App. The primary aim of the study described here is to examine improvement in glycaemic control of a new model of care employing MDMS for patients with complex T2DM attending a tertiary level outpatient service. A two-group, 12-month, pilot pragmatic randomised control trial will recruit 44 T2DM patients. The control group will receive routine care. The intervention group will be supported by the MDMS enabling the participants to potentially better self-manage their diabetes, and the endocrinologists to remotely monitor BGL and to interact with patients through a variety of eHealth modalities. Intervention participants will be encouraged to complete relevant pathology tests, and report on current diabetes management through an online questionnaire. Using this information, the endocrinologist may choose to reschedule the appointment or substitute it with a telephone or video-consultation. This pilot study will guide the conduct of a large-scale study regarding the capacity for a new model of care. This model utilises multimodal eHealth strategies via the MDMS to primarily improve glycaemic control with secondary aims to improve patient experience, reduce reliance on physical clinics, and decrease service delivery cost.

## 1. Introduction

Diabetes mellitus is a chronic disease resulting from increased blood glucose concentration that can lead to various health-related complications. Achieving recommended clinical glycaemic and cardiovascular risk factor targets through varying combinations of self-management, multidisciplinary specialist team input and/or medications, prevents or delays the development of complications related to diabetes. The global prevalence of diabetes is rising –with estimates of one in 11 people (425 million, 20–79 years) in 2017 projected to increase to one in 10 people (629 million, 20–79 years) in 2045 [1]. This increase in diabetes prevalence is mainly driven by Type 2 Diabetes (T2DM) (87–91% of diabetes prevalence in high income countries) and is related to increasing urbanisation, an ageing population and poor lifestyle choices [2]. Despite established evidence that suggests reductions in blood glucose marker (glycated haemoglobin-HbA1c) result in significant risk reductions of diabetes-related complications, only about 50% of people diagnosed with diabetes achieve recommended clinical targets [3]. This is concerning as the majority of diabetes burden is due to the treatment of complications. In Australia during 2015–2016, there were 980,000 hospitalisations related to T2DM comprising 10% of all hospitalisations, with national rates for diabetes-related potentially preventable hospitalisations being estimated to be as high as 180 per 100,000 people [3]. These figures underline the financial challenges faced by healthcare providers.

Given the challenges, continuation of conventional health service delivery approaches for T2DM (which are not optimal) will require dramatic and potentially unaffordable expansion of current services to meet the increasing demands of diabetes care. Moreover, health care has not been able to fully meet the transition requirements from an early 20th century acute medical conditions-based focus, to the current one with a high prevalence of chronic diseases. In a context of workforce shortages and high costs, a review of the models of outpatient diabetes care and support provided to people with T2DM is essential. A new model should be responsive and could interact with patients outside of their intended, routine, healthcare provider visits. Ideally, the model should incorporate crucial elements of the chronic care model [4] without incurring additional health-related costs. In this context, disruptive technologies like eHealth could redesign conventional approaches to outpatient care. WHO defines eHealth as ‘the use of information and communication technologies for health’ [5]. eHealth could potentially provide high quality, effective, efficient, and organised healthcare, that aims to support patient self-management in the community [6,7].

There is conflicting evidence regarding eHealth approaches and improved clinical outcomes in T2DM [8,9,10]. The eHealth technologies mostly employed are mobile applications, cloud-connected remote monitoring, patient and clinician decision support, clinician data management platforms, and telehealth [11]. Many eHealth interventions (e.g., mobile Apps for diabetes) offer glucose, food and insulin diaries with little perceived value in return which could lead to sub-optimal user-engagement [11,12]. Interventions that provide no clinical feedback are less likely to have significant reductions in HbA1c or as is the case of stand-alone text-messaging interventions, likely to have smaller effect in HbA1c reduction [13,14]. Mobile Apps for diabetes are largely unregulated with concerns for safety and there is minimal input from the stakeholders in the App development process which might affect use [15]. Patients could be offered various eHealth technologies as a combined package, with their actual use depending on their needs at a particular instance. Clinical data generated thus should be translated to useful information for the right person at the right time with minimal healthcare provider burden. Another challenge has been the integration of these new technologies into routine clinical care at scale because of a lack of infrastructure. A suggested approach is to offer a hybrid of eHealth solutions and conventional care to enable the gradual development of requisite infrastructure [16]. To our knowledge there is no published studies in Australia on hybrid models of care incorporating various eHealth interventions—to increase the efficiency of outpatient specialist diabetes service.

Considering the potential of eHealth technologies and the compelling need to reform diabetes care delivery, we designed an innovative model of specialist outpatient diabetes care utilising the Mobile Diabetes Management System (MDMS). The MDMS-enabled model of specialist outpatient diabetes care further described in the ‘Methods’ section incorporates the following elements: (1) targeting of intensive MDMS use to patients with suboptimal glycaemic control; (2) short or long-term remote monitoring of blood glucose level (BGL) and insulin dosage based on clinical need; (3) optional automated text-messaging feedback to patients based on BGL and self-monitoring frequency; (4) periodic patient self-report; and (5) substitution of conventional in-person follow-up consultations with telephone or video consultations. We hypothesise that these elements are likely to target patients at the highest risk of developing diabetes-related complications by improving clinical outcomes through better self-management at similar or lower costs. We aim to examine if a new model of care for complex T2DM patients attending a metropolitan specialist outpatient diabetes service improves glycaemic control at lower or similar costs to routine care.

## 2. Methods

### 2.1. Setting

Patients will be recruited from the specialist outpatient diabetes clinic at the Princess Alexandra Hospital (PAH), Brisbane, Australia.

### 2.2. Current Model of Outpatient Specialist Diabetes Service

The current model of outpatient T2DM diabetes care at a tertiary hospital in Australia entails regular outpatient clinic visits to access a multidisciplinary team led by an endocrinologist. The patients are usually referred by their general practitioner (GP). Specialist clinics, in general, have a mismatch between high service demand (referrals) and service capacity resulting in unacceptable waitlists. At a clinic visit, the patients are checked-in by the reception staff, and after a variable waiting period of 20–40 min, they are seen by an endocrinologist. The endocrinologist reviews previous clinical records and documents current status based on history from the patients or carers, BGL data, pathology reports and associated allied health reviews. BGL data is accessed through patient-provided paper records or data dumps from a variety of commercial glucose meters. This process can be unreliable and inefficient. The endocrinologist requests further allied health reviews and/or pathology testing as required. The interval between visits is determined by the endocrinologist based on glycaemic control, comorbidities and appointment availability. This interval can vary between 4 weeks to 6 months. The endocrinologist sends a letter to the patient’s GP regarding the outcome of each visit. Patients might access care from their GP in-between specialist visits if required. Clinic visits may involve patients travelling long distances, and waiting prolonged times at the clinic, requiring, for some, carer support and absence from their workplace. Patients who achieve a stable HbA1c < 64 mmol/mol (NGSP unit: 8%) are usually discharged back to the care of the GP with a management plan. Patients with sub-optimal glycaemic control (HbA1c usually over 64 mmol/mol (NGSP unit: 8%)) requiring insulin initiation or titration are often referred to a resource-intensive insulin dose adjustment (IDA) service. This process has been described in detail elsewhere [17]. Briefly, the IDA service is performed by a diabetes health professional, often a credentialled diabetes educator (CDE). The CDE obtains a combination of fasting, pre-meal and post-prandial BGL data over the telephone, and provides dose adjustment and general advice, once or twice weekly, over a period of 4–6 weeks. Most patients achieve individual target glucose levels in this time period. If not, either they are continued in the IDA service or their scheduled clinic appointment is advanced to identify other variables that may influence glycaemic management. The process of IDA is inefficient, mainly related to the issues of (1) unavailability of the CDE and the patient to review data over the phone simultaneously, (2) problems with transferability of BGL data to the CDE, (3) patient’s BGL monitoring frequency below recommendations, and (4) transcription errors using conventional approaches of data recording.

### 2.3. Description of MDMS

**Figure 1:**
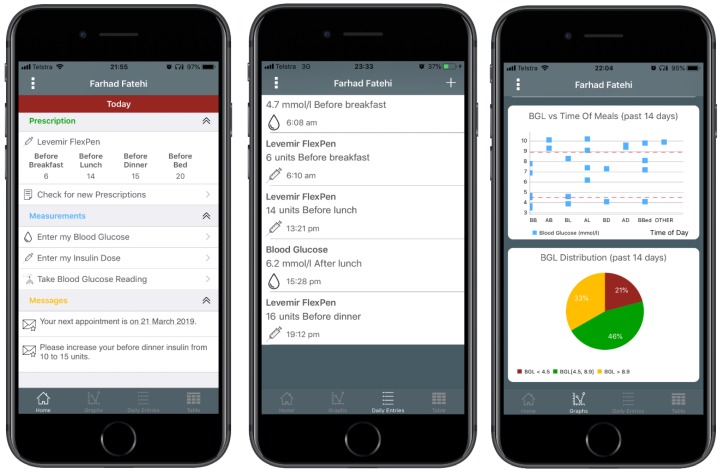
Screenshots of Mobile App iOS version. The app interface screenshot on the left depicts current insulin dosages and an option of downloading the latest changes made by the healthcare provider (HCP). Messages sent by the HCP are shown on the bottom. The middle screenshot displays insulin and blood glucose record whereas the right side shows the various graphical formats for easy visualisation of blood glucose data.

**Figure 2:**
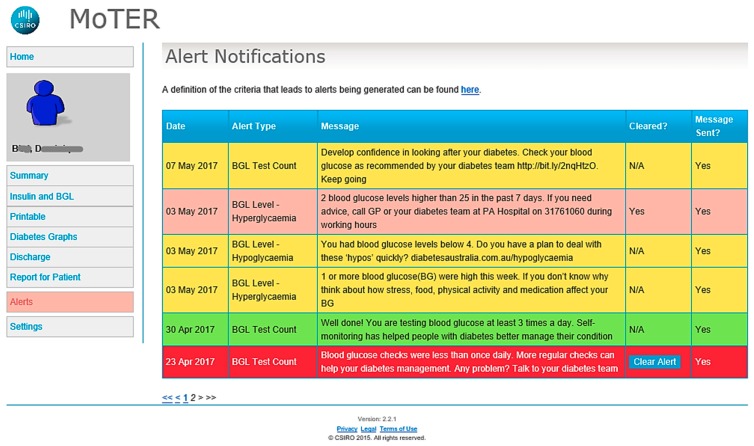
Screenshot of clinician portal showing an example of automated text-messages (accessed by clicking on the alerts tab on the left) sent to the patient based on blood glucose level and testing frequency which are colour coded for easy triaging by the clinician. Clicking on the home tab to the left of the screenshot will display a summary of individual patient details arranged according to the number of red alerts (text-messages).

In partnership with the Australian e-Health Research Centre, CSIRO (Commonwealth Scientific and Industrial Research Organisation), and the Department of Diabetes and Endocrinology at PAH, the Centre for Online Health, a sub-centre of the Centre for Health Services Research, The University of Queensland, developed MDMS to support the transformation of the provision of specialist diabetes care. This system is modelled on a highly successful CSIRO platform that supports in-home cardiac rehabilitation leading to improvements in adherence and clinical outcomes [18]. The system has been modified to enable real-time monitoring of BGLs of patients with diabetes and to support patient care. The system comprises an App for iOS-(Apple) (Figure 1) and Android-based smartphones, and a clinician portal. The mobile App enables patients to use a Bluetooth-enabled glucose meter (Accu-Chek^®^ Aviva Connect, Roche Diagnostics GmbH, Basel, Switzerland) to upload their BGL readings automatically to the clinician portal. The mobile App enables a review of BGL and their trends. The App also provides an insulin diary that allows patients to manually enter the dose and time of their insulin injections, along with a free text comment for each dose (e.g., “before dinner”). The data are subsequently transmitted and uploaded to the clinician portal via the Internet. Automated text-messages are triggered when patients have severely low or high BGL values (<2.5 and ≥25 mmol/L). All patients also receive regular automated text-messages based on BGL values and the frequency of BGL self-monitoring twice a week. Positive feedback messages are sent if these parameters are within clinically recommended targets. If parameters are outside of targets, the messages serve as prompts to check BGLs as recommended, to consider reasons for out of range BGL measurements and to seek medical advice if required. The messages also contain links to the national Diabetes Australia website for additional information regarding self-management of diabetes (Figure 2). Patients can choose to not receive these messages. The clinician portal presents the data uploaded by patients in graphical and tabular formats for the CDEs and endocrinologists to monitor and manage a patient’s condition. Through the portal, the clinicians can review patients’ BGL data and insulin dosages and send messages to the patients’ mobile phones. The home page of the portal displays a summary of individual glycaemic status—where patients are listed in order of the number of triggered automated text-messages. This is designed to help the prioritisation of follow-ups of patients. The process of home page review can be customised depending on desired outcomes and local resources. We have obtained patient and clinician feedback on the MDMS through a proof of concept (*n* = 9) and feasibility trial (*n* = 19) enrolling participants with T2DM, to enhance the platform [19]. This facilitated enhancements to the MDMS. The MDMS is being utilised in this RCT.

### 2.4. Study Design

**Figure 3:**
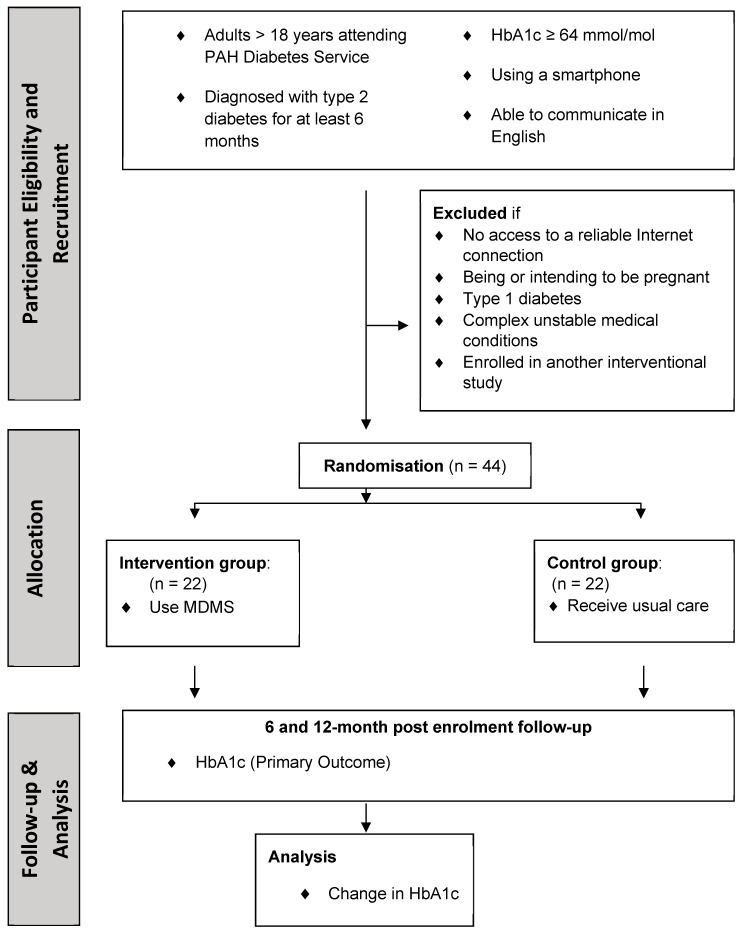
Study Design. PAH—Princess Alexandra Hospital, HbA1c—Glycated Haemoglobin; MDMS—Mobile Diabetes Management System.

The study will be a 2-group pilot pragmatic randomised controlled trial (RCT) with a 12-month follow-up period (Figure 3). Adults with T2DM attending the PAH outpatient diabetes service with suboptimal glycaemic control defined as HbA1c ≥ 64 mmol/mol (NGSP unit: 8%) will be recruited. This cut-off was chosen as patients with stable HbA1c < 64 mmol/mol (NGSP unit: 8%) are usually promptly discharged back to primary care, and because the greatest need is for patients who find it difficult to achieve management targets. Patients who meet the eligibility criteria will be included after consenting. Subsequently, they will be seen by the endocrinologist at a clinic visit when a 12-month diabetes management plan will be developed. The participants will then be randomised using the REDCap (Research Electronic Data Capture) software [20] and stratified based on (1) insulin usage—on insulin or not, and (2) new or pre-existing users of the PAH diabetes service.

### 2.5. Participants

Forty-four patients with T2DM attending the PAH outpatient diabetes service will be recruited. The inclusion criteria are (1) T2DM pre-existing for at least 6 months; (2) age > 18 years; (3) HbA1c ≥ 64 mmol/mol (NGSP unit 8%) (performed in the 4 weeks prior to enrolment); (4) using a smartphone daily; and (5) able to read and write in English.

Exclusion criteria are (1) having no access to a reliable Internet connection (reliable Internet connection is defined as access to 4G, 3G or Wi-Fi connection), (2) being or intending to be pregnant, (3) having type 1 diabetes, (4) having complex unstable medical conditions (i.e., requiring frequent hospital admissions unrelated to diabetes glycaemic management; being on varying doses of other medications such as glucocorticoids which can impact on glycaemic control; and having had bariatric surgery in the previous one year) or (5) being enrolled in another interventional study.

### 2.6. Outcomes

The primary outcome measure will be changed in HbA1c. Secondary outcome measures will be (1) clinical outcomes—percentage of participants achieving target HbA1c, change in blood pressure, lipid profile, body mass index and mean self-reported number of hypoglycaemia events in the 12th month follow up; (2) patient satisfaction; (3) quality-of-life assessed using the AQOL-8D (Assessment of Quality-of-Life) questionnaire [21]; (4) CDE satisfaction; and (5) rates of ‘did not attend’ clinic appointments.

For the participants in the intervention group, patient acceptance will be assessed using the Service User Technology Acceptability Questionnaire (SUTAQ) [22]. The type and number of substituted clinics and costs from both patient and healthcare provider (hospital) will be reported.

### 2.7. Sample Size and Power Calculation

A sample size estimation to show a mean difference of HbA1c of 0.5% between the two groups with a power of 0.8 and alpha of 0.05 using the Student’s *t*-test revealed that we would require 124 participants (SD of 1.4) in each group. This trial is a pilot which will help guide the conduct of a larger multi-centre trial regarding (1) identifying ease and rate of patient recruitment and (2) evaluating the financial and logistic feasibility of a full-scale study which will help us secure funds from relevant funding bodies. We will recruit 44 patients with a planned 1:1 randomisation between groups and allowing for a 10% attrition rate. This sample size for the pilot study was chosen based on a previous study done in a similar setting [23].

### 2.8. Ethics

Human Research Ethics Committee approval has been obtained through the Metro South Health Human Research Ethics Committee (Ref HREC/16/QPAH/373) and registered prospectively with Australia New Zealand Clinical Trials Registry (ACTRN 12617000980336).

### 2.9. Control Group

Participants in the control group will be managed according to the current model of care described in Section 2.2. The control group will be provided with new standard glucose meters if their current glucose meter is more than two years old.

### 2.10. Intervention Group

Participants in the intervention group will be managed through the new model of care utilising the MDMS. Following allocation to the intervention group, the project officer will install and demonstrate the MDMS smartphone App on the participants’ phone. They will then provide and train the participants in the use of the Roche glucose meter (Accu-Chek Aviva Connect), which connects via Bluetooth to the App to send BGL automatically to the web-portal daily. The project officer will set up access to the Queensland Health Telehealth Portal for video-consultation; and demonstrate how to complete the MDMS Preclinic Visit Questionnaire (which addresses all essential aspects of diabetes self-management, including diet, physical activity, medication adherence, diabetes-related stress and reduction of diabetes-related risk). The Queensland Health Telehealth Portal provides a secure way to videoconference with the clinician from within a web browser on the patient’s computer or through a separate App on their tablet or smartphone [24].

For 12 months, the participants will use the MDMS. This will involve (1) monitoring and automatic upload of BGL to the web-portal using the Accu-Chek Aviva Connect meter and smartphone App (2) if on insulin, entering insulin doses into the App and (3) receiving optional automated text-messages based on BGL value and testing frequency. Three weeks before a scheduled clinic appointment, the participants will receive a reminder by the research CDE to complete their regular clinical data collection—pathology tests, blood pressure and weight measured by a GP or at a pharmacy. The research CDE will inform the outcome of the pathology results to the participant once it is available. Two weeks before the scheduled clinic appointment they are sent a link to a Preclinic Visit Questionnaire to enter blood pressure and weight, to answer questions on healthy lifestyle management and advise the treating team of any diabetes-related concerns.

One week before the scheduled appointment with the participant, the endocrinologist will review the BGL, uploaded participant data, and pathology tests to decide the need for an appointment at this time. Based on this review the endocrinologist may either postpone, substitute the in-person appointment with a remote consultation (telephone or videoconference into the participant’s home) or leave the appointment unchanged. The participants will be notified of this plan via a text-message with details to call back if they have an alternate preference. A suitable alternative will then be discussed and agreed upon by the participant and the treating team. For a video-conference appointment, the participant will be sent an email with the web-link if using a computer or a text-message with the dial-in number if using the Telehealth Portal App. If the appointment is postponed, a letter will be sent to the participant and their GP regarding diabetes management status. At 12 months post recruitment, all participants will attend the clinic for an in-person consultation with an endocrinologist and CDE.

Intervention participants will be informed that the web-portal data will not be reviewed at times other than just before and at scheduled appointments unless enrolled in the IDA service or if specifically requested by the participant.

### 2.11. Data Collection (Table 1)

Study data will be collected and managed using REDCap electronic data capture tools hosted at the University of Queensland. REDCap is a secure, web-based application designed to support data capture for research studies, providing: (1) an intuitive interface for validated data entry; (2) audit trails for tracking data manipulation and export procedures; (3) automated export procedures for seamless data downloads to common statistical packages; and (4) procedures for importing data from external sources.

### 2.12. Analysis

Statistical analysis will be performed using SPSS (IBM Corp. Released 2017. IBM SPSS Statistics for Windows, Version 25.0. Armonk, NY, USA). Primary analyses will be based on ‘intention to treat’. A per-protocol analysis will also be conducted. Differences between groups at baseline and the endpoint will be compared using either the Student’s t-test or Mann–Whitney U-test as appropriate. Differences in repeated measurements will be compared using the Mixed-effects Model, adjusting for any significant explanatory variables. A *p* value of less than 0.05 will be considered statistically significant. A subgroup analysis of the participants new to the service and on insulin shall be carried out. Participants who are on insulin might require dose adjustments and thus might use the MDMS to interact with healthcare providers more often. Participants being referred to the specialist diabetes service might have different characteristics to the diabetes patients, already known to the service, with sub-optimal control despite specialist input.

Cost-estimations for delivery of the intervention will be performed at 12 months. The research costs will be excluded from this estimate. A full economic analysis is beyond the scope of this trial given the small number of trial participants.

## 3. Conclusions

The paper describes the protocol of a pilot pragmatic RCT utilising the MDMS in an Australian metropolitan area. The MDMS has enabled the design of a new model of specialist outpatient diabetes care that potentially allows greater patient self-management, increased efficiency with appointments only required when necessary and is a stepwise progression from the previous proof of concept study utilising the MDMS which showed promising levels of patient adherence and usability. We aim to examine various eHealth strategies as a multi-modal package (pragmatic approach) to better understand their effect on clinical outcomes, stakeholder satisfaction and, healthcare provider and participant costs. This pilot study will inform recruitment yield, retention and acceptability from people with T2DM and clinicians to guide the conduct of a large pragmatic RCT assessing the benefits of MDMS for specialist outpatient care. For eHealth interventions to be incorporated into models of diabetes care and adopted at scale, changes need to occur in entrenched clinician culture, infrastructure, policy and medical reimbursements. To facilitate this change, healthcare policy makers and administrators need evidence to fund implementation studies and subsequent integration into routine care. We believe this pilot trial will inform the redesign of healthcare delivery for chronic diseases such as diabetes in an Australian health context. The findings of this study might be useful for other similar healthcare settings.

## Figures and Tables

**Table 1:** Schedule of Enrolment, Intervention and Assessments.

**MDMS**	**STUDY PERIOD—12 Months**			
	Consent	Allocation	Post-allocation	Close-out
TIMEPOINT	Baseline		6 months	12 months
ENROLMENT:				
Eligibility screen	X			
Informed consent	X			
Allocation		X		
INTERVENTION: MDMS		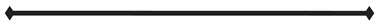		
ASSESSMENTS:				
HbA1c ^#^	X		X	X
Blood pressure, Body Mass Index, Lipid profile		X	X	X
Patient satisfaction survey and Diabetes Distress Scale		X	X	X
CDE satisfaction survey				X
Health-related Quality-of-Life—Assessment of Quality-of-Life (AQoL-8D) questionnaire		X	X	X
Self-reported hypoglycaemic events survey			X	X
Clinic Attendance Rates		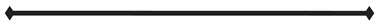		
**Other Assessments**				
Patient Acceptability Survey—Intervention group (SUTAQ) ^			X	X
Clinic Appointment types		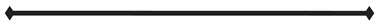		
Cost-estimations for delivery of intervention		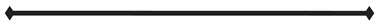		

MDMS—Mobile Diabetes Management System; ^#^ HbA1c also measured at three months; CDE—Credentialled Diabetes Educator; ^ SUTAQ—Service User Technology Acceptability Questionnaire.
